# The potential of exosomal biomarkers: Revolutionizing Parkinson's disease: How do they influence pathogenesis, diagnosis, and therapeutic strategies?

**DOI:** 10.3934/Neuroscience.2024023

**Published:** 2024-09-23

**Authors:** Naeimeh Akbari-Gharalari, Maryam Ghahremani-Nasab, Roya Naderi, Leila Chodari, Farshad Nezhadshahmohammad

**Affiliations:** 1 Department of Physiology, School of Medicine, Urmia University of Medical Sciences, Urmia, Iran; 2 Department of Tissue Engineering, Faculty of Advanced Medical Sciences, Tabriz University of Medical Sciences, Tabriz, Iran; 3 Neurophysiology Research Center, Cellular and Molecular Medicine Research Institute, Urmia University of Medical Sciences, Urmia, Iran; 4 Department of Mining Engineering, Faculty of Engineering, Urmia University, Urmia, Iran

**Keywords:** Parkinson's disease, exosomal biomarker, pathogenesis, diagnosis, treatment

## Abstract

Parkinson's disease (PD) is characterized by the pathological accumulation of α-synuclein, which has driven extensive research into the role of exosomes in disease mechanisms. Exosomes are nanoscale vesicles enriched with proteins, RNA, and lipids that facilitate critical intercellular communication processes. Recent studies have elucidated the role of exosomes in transmitting misfolded proteins among neurons, which significantly impacts the progression of PD. The presence of disease-associated exosomes in cerebrospinal fluid and blood highlights their substantial diagnostic potential for PD. Specifically, exosomes derived from the central nervous system (CNS) have emerged as promising biomarkers because of their ability to accurately reflect pathological states. Furthermore, the isolation of exosomes from distinct brain cell types allows the identification of precise biomarkers, increasing diagnostic specificity and accuracy. In addition to being useful for diagnostics, exosomes hold therapeutic promise given their ability to cross the blood–brain barrier (BBB) and selectively modulate their cargo. These findings suggest that these materials could be used as delivery systems for therapeutic drugs for the treatment of neurodegenerative diseases. This review comprehensively examines the multifaceted roles of exosomes in PD pathogenesis, diagnosis, and treatment. It also addresses the associated clinical challenges and underscores the urgent need for further research and development to fully leverage exosome-based strategies in PD management.

## 1. Introduction

Parkinson's disease (PD) is a prevalent neurodegenerative disorder that affected more than 9.4 million people globally in 2020, a significant increase from the previously reported 6 million individuals in 2016 [1],[2]. This shift in epidemiology has been observed worldwide. Various environmental factors, including pesticide and chemical exposure, as well as high dairy product consumption, have been linked to an elevated risk of PD. Conversely, certain behaviors, such as regular physical activity, caffeine consumption, smoking, and the use of specific medications such as ibuprofen and statins are associated with a reduced risk of PD [3]. Genetic factors are also important in the development of PD, with key genes such as leucine-rich repeat kinase 2 (LRRK2), the alpha-synuclein gene (SNCA), Parkin, DJ-1, PTEN-induced kinase 1 (PINK1), and glucocerebrosidase (GBA) being major contributors to its pathogenesis [4].

PD is characterized by the progressive degeneration of dopaminergic neurons within the substantia nigra compacta and the accumulation of misfolded α-synuclein (α-syn) protein within the cytoplasm of surviving neurons in this region. Braak's classification divides PD pathological progression into six distinct stages. Stages 1 and 2 signify a presymptomatic phase during which misfolded α-syn is localized to specific areas, such as the olfactory bulb, pontine tegmentum, and medulla oblongata. As PD progresses to stages 3 and 4, the substantia nigra and other nuclei in the midbrain and basal forebrain are affected, which coincides with the onset of motor symptoms. Pathological changes are shown to spread throughout the telencephalic cortex in the next stages, at stages 5 and 6 [5],[6]. It is common practice to divide PD symptoms into motor and nonmotor categories. Bradykinesia, tremors, muscular stiffness, and postural instability are the main motor signs. Olfactory dysfunction, constipation, rapid eye movement sleep behavior disorder (RBD), cognitive decline, sadness, and anxiety are examples of nonmotor symptoms [7]. The olfactory bulb, dorsal vagal nucleus, and locus coeruleus are among the brain regions affected in the early stages of PD and may be predictive indicators of future risk for the disease. As a result, the Braak hypothesis suggests that these nonmotor symptoms may appear before motor symptoms [8].

Early signs of PD are not very different from those of atypical Parkinsonian syndrome (APS). These include dementia with Lewy bodies (DLB), corticobasal degeneration (CBD), frontotemporal dementia (FTD), multiple system atrophy (MSA), progressive supranuclear palsy (PSP), and some rare diseases. As such, reaching a conclusive diagnosis becomes difficult. This comprises meeting at least two corroborating requirements and having bradykinesia in addition to stiffness or rest tremors. These additional requirements might include the development of levodopa-induced dyskinesia and a notable improvement in dopaminergic therapy, all while ruling out any obvious warning signs such as significant autonomic dysfunction during the first five years after disease onset [9]. However, it is worth noting that once classical motor symptoms manifest, a substantial toll has already been taken on dopaminergic neurons within the substantia nigra, with over half of them being lost, and dopamine concentrations within the striatum declining to less than 80% [10]. According to Braak's theory, the pathogenesis of PD could begin in olfactory organs and enteric nerves in the gastrointestinal tract and then spread to the substantia nigra, where dopaminergic neurons eventually die, and PD-related motor dysfunction manifests years or even decades later [11]. On the basis of this idea, the disease progresses significantly by the time motor symptoms manifest, and pathological changes occur before motor symptoms do. Consequently, it is essential to develop a unique biomarker that may identify PD early in life. Biomarkers that can mimic the early pathological alterations linked to PD have become necessary to aid in the early detection of this disease. According to this viewpoint, the secretion of exosomes by certain neuronal cells appears to be a viable pathway for identifying prospective biomarkers. Exosomes play a role in communication between interneurons and neurons in glia, as recent studies have shown. They act as carriers of misfolded α-syn from parent cells, which spreads illness between cells [12],[13]. Several studies have shown that exosomes produced by the central nervous system (CNS) can cross the extremely strong blood–brain barrier (BBB). By utilizing certain neural markers, it is possible to separate these exosomes from serum or plasma, providing a window for the identification of brain disease. This discovery offers a novel approach to the early detection of PD and has great potential for the noninvasive examination of brain biomarkers [14],[15].

The current therapeutic approaches for PD lack curative effects, despite the availability of pharmaceuticals that can alleviate motor symptoms, which may, however, lead to adverse effects as the disease progresses. These findings underscore the urgent need to explore novel pharmaceuticals or methodologies for managing PD. Although many CNS-targeted drugs fail in clinical trials because of their inability to penetrate the BBB, exosomes and nanosized vesicles possess the unique ability to traverse the BBB, making them promising candidates for drug delivery [16]. Compared with free dopamine, exosomes isolated from human blood and loaded with dopamine can breach the BBB and deliver dopamine to the brain, demonstrating enhanced therapeutic efficacy and reduced toxicity [17]. Additionally, exosomes engineered to carry catalase, an antioxidant, have been shown to mitigate neural inflammation and enhance neural viability in PD models [18]. Moreover, exosomes can transport small interfering RNAs (siRNAs) that target α-syn, reducing its levels and alleviating PD symptoms [19]. Mesenchymal stem cell (MSC)-derived exosomes have also shown promise in protecting dopaminergic neurons and reducing neuroinflammation in PD models through the delivery of beneficial microRNAs (miRNAs) [20]. Despite the progress made, obstacles persist, including the need for more efficient delivery mechanisms, exploration of potential negative impacts, and identification of the best cellular origin for exosomes. Nonetheless, exosomes offer a promising avenue for PD treatment and hold potential for various other neurodegenerative conditions.

## 2. Exosome features

Exosomes are small membrane-enclosed intracellular vesicles that range in size from 30 to 150 nm. These microscopic structures facilitate the transportation of various cellular components, including lipids, proteins, and nucleic acids, to specific target cells by acting as conveyors for them [21]. The development of multivesicular bodies (MVBs) is thought to be the source of exosomes [22]. The cellular membrane folds inward during this genesis process, forming a cup-shaped structure that traps soluble molecules from the external environment and cell surface proteins. These vesicles then combine to produce early-sorting endosomes, which subsequently develop into late-sorting endosomes. Many vesicles known as intraluminal vesicles (ILVs) are formed as the membranes of these late-sorting endosomes continue to fold inward. Late-stage endosomes are referred to as MVBs. These MVBs have two possible fates—they can merge with lysosomes for degradation or fuse with the plasma membrane to release ILVs as exosomes [23],[24]. Exosomes are produced by numerous cell types, including neurons, MSCs, and immunological cells. Moreover, they are found in many other body fluids, such as blood, saliva, urine, and cerebrospinal fluid (CSF) [25]. Exosomes may carry a wide range of payloads, including particular proteins, lipids, and genetic material, most notably RNA and DNA [26]. Lipids found in exosomes are essential for both coordinating vesicle production and extracellular release as well as for preserving the structural integrity of the exosome membrane. Exosomal lipids are distinguished by their unique distribution between the inner and outer membranes. Studies have demonstrated that phosphatidylcholine and sphingolipids are primarily located in the outer membrane, while other lipid species are predominantly found in the inner membrane [27]. Two general categories may be used to classify the protein components of exosomes, namely common components and specialized components. Heat shock protein 70 (HSP70), heat shock protein 90 (HSP90), ALG2-interacting protein X (Alix), CD81, CD63, tumor susceptibility gene 101 (Tsg101), and membrane transport and fusion-related proteins such as Rab GTPases are examples of common components. These proteins are essential for the production and secretion of exosomes [28]. On the other hand, certain elements are closely connected to the parent cells from whence they originated. For example, mast cell-derived exosomes containing markers such as CD86, MHC-II, intercellular adhesion molecule 1 (ICAM-1), and lymphocyte function-associated antigen 1 (LFA-1) can stimulate the growth of B and T cells [29]. Furthermore, PD-L1-containing melanoma-derived exosomes suppress CD8+ T-cell antitumor activity in vivo, thereby encouraging tumor expansion [30]. Additionally, exosomes act as transporters of genetic material, including DNA and RNA, which may be passed from one cell to another and regulate the expression of certain genes in the recipient cell [31],[32].

## 3. Feasibility and challenges of exosome-based therapies

Evaluating the feasibility and practicality of exosome separation methods for clinical applications involves examining both their efficiency and operational challenges. Ultracentrifugation is widely regarded as the gold standard because of its effectiveness in isolating exosomes, but it is hindered by high costs, complexity, and the risk of damaging exosome structures. Size exclusion chromatography (SEC) offers high purity and preserves exosome integrity, although its time-consuming process limits its scalability for clinical use [33]. In contrast, filtration and precipitation methods are more cost-effective and faster but often result in lower purity because of contamination from microvesicles and lipoproteins. Microfluidic techniques show promise with their rapid processing, high sensitivity, and small sample volume requirements but are not yet optimized for large-scale or clinical applications [34].

In addition to separation challenges, the use of exosomes as therapeutic vectors presents several disadvantages. One major issue is the incomplete understanding of how exosomes mediate their therapeutic effects, which complicates the optimization and predictability of such therapies. Variability in exosome content on the basis of their source and isolation conditions can also affect the consistency and efficacy of treatments [35]. Moreover, contamination with other cellular components during isolation may impact the safety and effectiveness of exosome therapies. Scaling up production for clinical use introduces further complications, as current isolation and purification methods may not be easily adapted for large-scale processes [36]. Addressing these challenges and regulatory concerns through detailed studies is crucial for advancing exosome-based therapies and maximizing their potential for clinical applications.

## 4. Exosomes in PD: Pathogenesis

Exosomes play a major role as mediators in promoting intercellular communication and are highly relevant in many disease processes, including the etiology of PD [37]. Misfolded α-syn builds up in neurons and is a hallmark of PD. Notably, misfolded α-syn from damaged neurons is transferred to healthy neurons via exosomes, which in turn causes protein aggregation and cell death. The autophagy–lysosome pathway (ALP) is consistently damaged in both rodent PD models and postmortem brain tissue of PD patients, indicating a close association between this phenomenon and autophagic impairment [38],[39]. Recent work by Georgia Minakaki and colleagues revealed that ALP malfunction causes an increase in the amount of α-syn in exosomes generated from neurons, which promotes α-syn transfer across cells in vivo [40]. Moreover, recipient cells internalize more α-syn oligomers encapsulated in exosomes than loose α-syn oligomers do [13],[41]. These findings highlight how crucial exosomes are in the spread of α-syn disease. According to research by Huang et al., when neural-derived exosomes from PD patients are injected into the striatum of mice, they can cause dopaminergic neuron degeneration and consequent motor impairments [42]. Exosome-induced α-syn aggregation can be further aggravated by inflammatory stimuli, hence increasing α-syn toxicity [13]. Research carried out by Han and colleagues revealed increased concentrations of TNF-α and IL-1β in the serum exosomes of PD patients. Moreover, injecting these serum exosomes into the mouse striatum resulted in the accumulation of α-syn, the activation of microglia, and the degeneration of dopaminergic neurons, providing strong evidence that damage to dopaminergic neurons is amplified by exosome-mediated inflammatory cytokine transport [43].

## 5. Exosomes in PD: Diagnosis

Recently, exosomes—tiny, membrane-enclosed vesicles released by cells—have gained attention as promising options for enhancing PD detection. In this context, exosomal RNA species such as miRNAs and long noncoding RNAs (lncRNAs), as well as exosomal proteins such as α-syn, DJ-1, LRRK2, and tau have attracted much attention due to their potential as useful diagnostic markers.

### 5.1. Proteins

#### 5.1.1 α-Syn

In the setting of PD, α-syn, which is normally present in a highly soluble and unfolded state, changes and aggregates into insoluble filaments. This aggregate, commonly called Lewy bodies, is a pathogenic feature that distinguishes PD from other illnesses grouped together as α-synucleinopathies. According to recent studies, misfolded α-syn can potentially have prion-like characteristics. Recently, exosomes have been identified as possible causes of this disease process. According to recent studies, misfolded α-syn can be contained in exosomes, secreted by neuronal cells into the extracellular matrix, and then utilized by neighboring neurons. This process has a significant effect on how PD develops. As a result, the identification of α-syn in exosomes has attracted interest as a potential diagnostic tool for PD [44],[45]. To further elucidate the potential of exosomal α-syn as a diagnostic marker for PD, this review explores studies conducted across three primary biofluids: CSF, blood, and saliva. Each of these sources will be examined in detail, and their roles, diagnostic potential, and associated challenges will be discussed:

##### 5.1.1.1 α-Syn in CSF exosomes

Recent research into the diagnostic potential of exosomes, particularly CSF, in PD has highlighted their diagnostic potential. Stuendl et al. demonstrated that α-syn levels in CSF exosomes are lower in the early stages of PD than in healthy controls, suggesting that α-syn in CSF exosomes could serve as a valuable biomarker for PD. They also identified a pathogenic form of α-syn in these exosomes, which might initiate the aggregation of soluble α-syn in target cells, contributing to disease progression [46]. However, CSF collection remains invasive and challenging. In contrast, in healthy controls, α-syn levels in CSF exosomes are relatively stable and not associated with disease pathology. Further research involving the administration of CSF exosomes from PD patients to healthy mice revealed PD-like symptoms, including motor and nonmotor impairments and α-syn aggregation in the brain. This study supports the idea that CSF exosomes can propagate α-syn pathology and exacerbate PD symptoms [47]. Moreover, multiplex analysis of single exosomes from the CSF of Parkinson's disease dementia (PDD) patients via solid-state technology revealed various surface markers, such as CD9, CD63, and CD81. A notable reduction in ApoE+ exosomes was found in PDD patients compared with healthy controls, whereas α-syn was not detected on the exosome surface [48]. In contrast, recent studies have explored the role of plasma exosomes in PD. They reported that while α-syn levels are lower in CSF, plasma exosomal α-syn levels are significantly higher in PD patients. This increase suggests that α-syn is released from the CNS into the peripheral blood. Plasma exosomal α-syn correlates with disease severity and shows diagnostic sensitivity and specificity comparable to those of CSF α-syn. These insights highlight the potential of plasma exosomal α-syn as a less invasive biomarker for PD, which could be a focus of future diagnostic and therapeutic research [49].

##### 5.1.1.2 α-Syn in blood exosomes

Exosomes derived from the CNS present in the blood, especially those carrying α-syn, offer new diagnostic opportunities for PD. L1 cell adhesion molecule (L1CAM), a CNS-expressed protein, is used to isolate neuronal exosomes from blood and links α-syn levels with disease severity and progression [49],[50]. Studies by Niu et al. [51] and Yan et al. [52] demonstrated that α-syn concentrations in neuron-derived exosomes from plasma samples are significantly higher in PD patients, including those in the prodromal phase, than in healthy controls [53]. This increased presence of α-syn in blood-derived exosomes makes it a promising biomarker for distinguishing PD from non-PD states. However, conflicting results have been reported in the literature. For example, Shim et al. [54] reported no significant differences in plasma exosomal α-syn levels between PD patients and healthy individuals, whereas Si and colleagues reported lower α-syn levels in CNS-derived exosomes from PD patients. These discrepancies highlight the impact of different isolation methods and biofluids on study outcomes. Despite these conflicting findings, recent studies support the potential of α-syn in blood exosomes as a valuable tool for diagnosing and monitoring PD. Research also indicates that serum levels of CNS-derived exosomal α-syn can be used to differentiate between PD patients and healthy controls and help identify different motor types within PD [55]. Furthermore, the identification of pathological α-syn forms through immunoblot analyses, which exhibit β-sheet-rich structures and fibrillary appearances, provides new insights into the mechanisms of neurodegenerative disorders [56]. Recent advancements suggest that α-syn in neuron-derived exosomes holds significant promise for early diagnosis and monitoring of disease progression in PD.

##### 5.1.1.3 α-Syn in saliva exosomes

Exosomes derived from saliva are increasingly being explored as promising noninvasive sources of biomarkers for PD. These small extracellular vesicles, which are secreted into the saliva, can provide valuable insights into the biochemical alterations associated with PD. Recent studies have investigated the presence and levels of α-syn within salivary exosomes to evaluate its potential as a diagnostic indicator for PD. One of the key findings from recent research is that salivary exosomes from PD patients exhibit significantly higher levels of oligomeric α-syn (α-synOlig) than those from healthy controls [57]. For example, a study analyzed saliva samples from 74 PD patients and 60 healthy controls and discovered that the levels of α-synOlig in PD patients were notably elevated. Additionally, the ratio of α-synOlig to total α-syn was significantly greater in PD patients than in healthy individuals. This increase in α-synOlig and the α-synOlig/α-synTotal ratio highlights the potential of these biomarkers in distinguishing PD from non-PD states. In contrast, healthy controls generally have lower levels of α-synOlig and a lower α-synOlig/α-synTotal ratio in their saliva. This discrepancy between PD patients and healthy individuals underscores the diagnostic potential of measuring these specific forms of α-syn in saliva. Notably, while total α-syn levels in saliva did not significantly differ between PD patients and healthy controls, the oligomeric form of α-syn was significantly increased in PD patients. These findings suggest that α-synOlig and the α-synOlig/α-synTotal ratio are more indicative of PD status than are total α-syn levels alone [58]. Moreover, the presence of specific markers such as Alix and CD9 in salivary exosomes further supports the potential of these vesicles as diagnostic tools. These markers indicate that the exosomes isolated from saliva are indeed of neuronal origin, which is relevant for PD diagnostics. The noninvasive nature of saliva collection provides a significant advantage over blood-based methods, which can be more invasive and subject to variability due to systemic factors. The ability to detect elevated levels of α-synOlig and the α-synOlig/α-synTotal ratio in salivary exosomes offers a more accessible and potentially reliable diagnostic approach for PD. However, it is important to note that there are conflicting results in the literature regarding salivary exosome and α-syn levels [59]. Some studies have reported elevated levels of α-syn, whereas others have reported reductions or no significant differences, indicating the need for standardized methodologies and further research to confirm these findings and establish their clinical relevance [60],[61].

#### 5.1.2 DJ-1

When exposed to oxidative stress, the antioxidative protein DJ-1 automatically oxidizes, protecting cellular components and coordinating gene expression to strengthen antioxidant defenses [62]. Owing to its connection to the processes underlying PD, researchers have investigated the possibility of using DJ-1 as a biomarker. Differences in DJ-1 levels have been detected in a variety of bodily fluids, such as urine [63], serum [64], plasma [65], and CSF [66]. For example, the levels of oxidized DJ-1 (OxiDJ-1) in the urine of PD patients were found to be significantly greater than those in the urine of non-PD controls by a factor of two [63]. In contrast, Zhao et al. examined plasma DJ-1 levels in both healthy controls and PD patients but reported no appreciable differences between the two groups [67]. However, when scientists separated neural-derived exosomes from plasma, they reported that PD patients had far greater quantities of DJ-1 than healthy controls did. Notably, exosomal DJ-1 derived from neuron-generated exosomes in plasma samples was strongly positively associated with α-syn in both individuals with PD and healthy controls; however, no association was found with disease progression. Notably, proteomic studies of urine exosomes have revealed the presence of DJ-1 [68],[69]. In one study, Dong Hwan Ho and colleagues compared the concentrations of DJ-1 in urine exosomes taken from both non-PD patients and PD patients to investigate the potential of urine exosomes as a diagnostic tool for PD. The results demonstrated that only male PD patients had an approximately 1.7-fold increase in DJ-1 protein, indicating that DJ-1 levels in urine exosomes may be a useful biomarker for male PD diagnosis. In general, DJ-1 is still not regarded as a frequently used biomarker. Although its diagnostic performance is moderate, its correlation with disease progression is still unknown. Moreover, the fundamental causes of sex disparities in DJ-1 levels are yet unknown. To address these issues, larger patient groups and further studies are needed [70].

#### 5.1.3 Tau

The stability of the microtubule bundle is maintained by tau, a microtubule-associated protein (MAP) that is present mostly in neuronal axons [71]. It is also important in tauopathies, one of which is Alzheimer's disease (AD) [72]. Additionally, a fascinating relationship has been found between the tau gene (MAPT) and vulnerability to intermittent PD, as reported by multiple studies [73]–[75]. When PD patients' tau protein levels were compared to those of healthy individuals, a postmortem examination of the human striatum revealed greater quantities of the protein [73]. Remarkably, the reduction in neurodegenerative histopathological markers indicated a significant decrease in the course of α-synucleinopathy with tau expression depletion [76]. This research investigated the potential use of tau inside neuron-derived exosomes from plasma samples as a marker for diagnosis. The mice were intracerebroventricularly injected with radiolabeled or unmarked tau, and the outcomes yielded significant new data. This study demonstrated that tau can be found within neuron-derived exosomes from plasma samples and can be transferred from the brain to the circulation. Crucially, rather than being on the surface of the exosomes, the tau linked with them was encapsulated primarily within them. These results were then applied to human participants, and the results of these studies revealed that PD patients had considerably greater quantities of tau inside neuron-derived exosomes from plasma samples than healthy controls did. On the other hand, compared with both PD patients and healthy controls, AD patients have much greater amounts of tau throughout their whole plasma [77]. Thus, the diagnostic potential of tau in the setting of PD deserves more research, especially in conjunction with other candidate proteins, such as α-syn.

#### 5.1.4 LRRK2

Most cases of PD are classified as idiopathic PD (iPD) because no environmental or genetic factors are responsible for 90% of cases. The remaining 10% of patients have a known genetic component and are likely to have a familial onset. Among those with familial onset, mutations in the LRRK2 gene are the most common cause of late-onset PD [78]. Surprisingly, researchers only discovered very recently, in 2004, that LRRK2 is the only gene most closely linked to PD [79]. Research has revealed that the most common missense mutation in LRRK2 results in elevated LRRK2 protein kinase activity, which is connected to the onset of PD [80]. The LRRK2 protein was detected in exosomes made from urine and CSF by Fraser and colleagues in 2013. However, LRRK2 levels in exosomes do not differ between PD patients and healthy controls [81]; in 2016, more investigations revealed important breakthroughs. The level of serine-1292-phosphorylated LRRK2 protein in urinary exosomes was found to be approximately five times greater in a preliminary cohort of PD patients carrying the G2019S-LRRK2 mutation than in healthy controls and PD patients without the G2019S-LRRK2 mutation [82]. These results suggest that elevated urine exosome levels of Ser(P)-1292 LRRK2 can function as a marker of LRRK2 mutation status and PD risk in individuals.

Nevertheless, even among those without specific mutations, LRRK2 has been shown to have a major influence on the pathogenesis of PD. Without these mutations, patients with iPD exhibit defective neuronal autophagy and LRRK2 protein overactivation. These malfunctions cause an aberrant build-up of α-syn, a protein that is strongly involved in PD progression [83]. Fraser et al. (2016) compared the amount of Ser-1292-phosphorylated LRRK2 (Ser(P)-1292 LRRK2) in biobanked urine samples to clinical information from patients with PD and control subjects. Our findings demonstrated that the levels of LRRK2 at Ser(P)-1292 in PD patients were significantly greater than those in control participants. Furthermore, a relationship was shown between the degree of cognitive impairment and Ser(P)-1292 LRRK2 levels in urine exosomes, highlighting the importance of this protein in the pathophysiology of PD [84]. Although the level of serine 1292-phosphorylated LRRK2 [Ser(P)-1292 LRRK2] has been shown to be elevated in the urine exosomes of PD patients, several unanswered questions remain that make it difficult to determine whether Ser(P)-1292 LRRK2 is a useful biomarker. To verify the usefulness of Ser(P)-1292 LRRK2 in exosomes for the diagnosis of PD or the evaluation of the severity of PD, including its association with both motor and nonmotor symptoms, more research is necessary.

### 5.2. RNAs

#### 5.2.1. miRNA

MiRNAs are a subclass of small noncoding RNA molecules that are typically between 21 and 24 nucleotides in length. These endogenous genes produce these molecules, which use the RNA interference (RNAi) mechanism to control mRNAs, hence performing regulatory roles within cells. MiRNAs play important roles in the epigenetics of many illnesses and are implicated in a broad range of cellular functions; they frequently show altered expression patterns in different disease states [85]. Interestingly, miRNAs are stable in biological fluids such as serum and plasma; they frequently attach to proteins or form vesicles, which protects them from breakdown. This feature has made miRNAs attractive biomarkers because they provide a solid basis for their detection in bodily fluids or vesicles.

Gui and colleagues produced a novel finding in a critical study from 2015 in which they found that individuals with PD and AD have miRNAs in their CSF exosomes [86]. Their research revealed that, compared with those in healthy controls, the expression of 16 miRNAs was elevated and that of 11 miRNAs was downregulated in PD patients. Independent sample sets were used to further validate these results. In CSF exosomes from PD patients, let-7 g-3p, miR-153, miR-10a-5p, and miR-409-3p were strongly overexpressed, whereas miR-1 and miR-19b-3p were substantially downregulated. Additionally, DIANA mirPath analysis was performed, which demonstrated that the miRNA patterns linked to PD were enriched in several important biological pathways. Furthermore, miR-409–3p has emerged as the most promising individual miRNA. Additionally, the combination of miR-153 and miR-409-3p significantly increased diagnostic accuracy.

Nie et al. thoroughly analyzed plasma exosomal miRNAs in individuals with PD via small RNA sequencing. Their research revealed a broad spectrum of changes in miRNA expression levels. Notably, they found that eight miRNAs displayed distinct expression patterns in PD patients. Among these, let-7e-5p was considerably increased in PD samples, suggesting its potential as a biomarker for PD [87]. Additionally, another study by Yao et al. identified miR-331–5p and miR-505 as potential biomarkers for PD [88].

Many studies have been carried out in an attempt to identify miRNA-based biomarkers for PD, and as a result, a number of miRNAs that show notable changes when associated with PD have been identified. However, disparities among these investigations have led to diverse conclusions [89]–[91]. Cao et al. conducted research to improve our knowledge of the potential of miRNAs as clinical biomarkers for PD to address this difference. To do this, they evaluated a collection of 24 miRNAs that have been identified as possible PD biomarkers in serum or plasma and determined whether they were appropriate for use in the setting of serum exosomes. Only three miRNAs produced consistent results in the present study; in serum exosomes from PD patients, miR-19b was downregulated, whereas miR-195 and miR-24 were overexpressed. The team then used the TargetScan program to investigate the verified gene targets linked to these miRNAs. These gene targets, which include ATP13A2/PARK9 (targeted by miR-24 and miR-195), LRRK2/PARK8 (targeted by miR-19b), and Parkin RBR E3 ubiquitin protein ligase (PARK2; targeted by miR-19b), are strongly correlated with various neurodegenerative processes, neuronal apoptosis, and regeneration that underlie PD [92]. Therefore, additional research must be conducted before exosomal miRNAs can be considered potential biomarkers for diagnostic use in the treatment of PD.

#### 5.2.2. lncRNA

**Figure 1.**
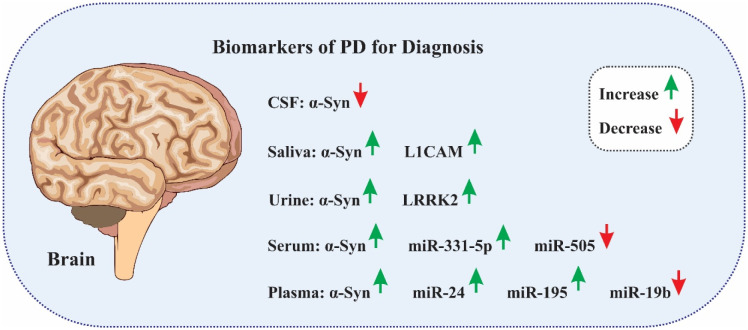
Biomarkers for PD diagnosis.

A family of RNA molecules known as lncRNAs is distinguished by their length, which usually surpasses 200 nucleotides. LncRNAs have been shown to be important in many biological processes, including RNA metabolism, stem cell maintenance and differentiation, transcriptional and translational regulation, epigenetic control, autophagy, apoptosis, and embryonic development [93]. Moreover, lncRNAs are closely linked to the pathophysiology of neurodegenerative diseases such as PD, AD, Huntington's disease (HD), and amyotrophic lateral sclerosis (ALS). Notably, lncRNAs are also essential for brain development, neuronal function, and maintenance [94]. The main topic of this debate is the use of exosomal lncRNAs as a PD diagnostic tool. In 2020, Zou et al. used a variety of clinical measures to evaluate the severity of PD in 93 patients and 85 control participants. Using antibody-coated superparamagnetic microbeads, they were able to separate exosomes carrying L1CAM from human plasma. The group then used microarray technology to examine the lncRNA profiles found in these exosomes [95]. Linc-POU3F3 expression was significantly upregulated in patients with PD, and this lncRNA had the greatest reliable detection density. Notably, β-glucocerebrosidase (GCase), a lysosomal enzyme produced by the GBA1 gene, has been linked to the stability of α-syn proteins and is essential for the pathophysiology of PD [96]. However, a strong relationship was found between plasma GCase activity, the severity of PD, and the amount of Linc-POU3F3 in L1CAM exosomes. However, PD patients and control participants were substantially separated when Linc-POU3F3 was used alone. When plasma GCase activity and LINC-POU3F3 and α-syn in L1CAM exosomes were taken into account, the separation was significantly improved. As a result, L1CAM exosomal Linc-POU3F3 shows promise as a PD diagnostic biomarker. Figure 1 shows the diagnostic biomarkers associated with PD.

## 6. Exosomal biomarkers: APS

Misfolded α-syn in oligodendrocytes is a characteristic of MSA, a deadly neurodegenerative disease with an unclear cause that manifests in adults [97],[98]. There are two primary forms of this gene: MSA-P, associated mostly with parkinsonism, and MSA-C, associated with cerebellar symptoms and both striatonigral degeneration (SND) and olivopontocerebellar atrophy (OPCA) [99]. Similar symptoms in the early stages of PD and MSA may occur, making diagnosis difficult [100],[101]. Dutta et al. used exosomes produced by neurons or oligodendrocytes and measurable in plasma to separate healthy people from PD patients and MSA patients. In their study, they used myelin oligodendrocyte glycoprotein (MOG) as a marker for the isolation of oligodendroglial exosomes, allowing them to specifically target exosomes derived from oligodendrocytes. In contrast to earlier studies, their data revealed that α-syn levels followed the order control < PD < MSA in both neuronal and oligodendroglial exosomes. Furthermore, the ratio of the α-syn concentration in neuronal exosomes to that in oligodendroglial exosomes was demonstrated to be an extremely sensitive biomarker for differentiating between MSA and PD [102],[103].

PSP is a variety of APSs frequently characterized by frontal lobe dysfunction, dysarthria, dysphagia, and vertical gaze palsy [104]. Distinguishing between PD and PSP, particularly in the beginning stages, might be challenging. However, Ida Manna et al. investigated the possibility of using miRNAs in serum exosomes as biomarkers to distinguish between PSP and PD. They reported that six miRNAs (miR-29a-3p, miR-199a–5p, miR–3p, miR-21–3p, miR-425–5p, and miR-483–5p) had excellent discriminatory power. These results suggest that combinations of exosomal miRNAs have the potential to improve diagnostic differentiation and should be further validated in larger cohorts to support clinical distinctions between PSP and PD [105].

Patients with DLB generally exhibit early-stage dementia and visual hallucinations at the same time. Extrapyramidal motor symptoms, similar to PD symptoms, typically appear simultaneously or quickly. Early in life, cognitive impairment usually starts after age 55 [106]. Visuospatial abnormalities, varied memory losses, and severe executive dysfunction are among the cognitive areas impacted in both DLB and PDD [107]. When DLB is diagnosed, the international consensus criteria take into account cognitive impairment that starts within a year after the onset of Parkinsonian motor symptoms or comes before them [108]. Conversely, cognitive impairment occurs in individuals with PDD when established PD is present [109]. These two illnesses show a great deal of convergence despite variations in the timing of motor and cognitive abnormalities as well as certain clinical variances [110]. It is difficult to differentiate between PD and DLB accurately since the two conditions have similar clinical signs and neuropathological alterations. One study suggested that CSF exosomal α-syn may be a biomarker that can differentiate between DLB patients and PD patients. Compared with CSF samples from PD patients and controls, DLB patients had fewer exosomes and decreased concentrations of exosomal α-syn [46].

Symptoms of CBD, a chronic, progressive neurological disease, include dystonia, postural abnormalities, dysfunction, and an asymmetric akinetic-rigid condition. The accumulation of abnormal tau proteins in glial cells and neurons is a hallmark of this disease pathogenesis. Initially, CBD was commonly misdiagnosed as Parkinson's disease or other degenerative conditions [111]. Recently, Meloni et al. examined exosomal α-syn and tau aggregates in neural-derived exosomes isolated from patient blood to distinguish PD from APS, such as CBD and PSP. Notably, PD patients had much greater amounts of exosomal oligomeric α-syn than APS patients did, whereas APS patients had significantly greater levels of exosomal tau aggregates than PD patients did [112].

By adding a significant number of new samples and combining them with their prior dataset, Jiang and colleagues carried out a thorough confirmation of their earlier findings in 2021. A total of 735 participants—including healthy control subjects and those in the PD, MSA, PSP, and CBD subgroups—were included in this combined study [113]. The idea that exosomal α-syn levels are helpful indicators for distinguishing PD from MSA and 4-repeat tauopathies is strongly supported by these findings. Furthermore, when used in conjunction with exosomal clusterin, this combination improves the ability to discriminate between 4-repeat tauopathies and PD. This fascinating discovery implies that future research should consider clusterin as one of the essential indicators in their diagnostic toolbox to distinguish between atypical Parkinsonian disorders.

This section highlights how important exosomes are in differentiating PD from other APSs. Additionally, the majority of research has been performed on exosomes with neural origins, but few comparative studies have investigated exosomes from other kinds of brain cells, such as neurons, oligodendrocytes, and astrocytes. Moreover, we propose a broader perspective on the potential analysis of exosomes originating from nigrostriatal dopaminergic neurons in future research, which could significantly advance the diagnosis of PD. Furthermore, given the diverse pathological protein profiles observed in APS, the diagnostic process for PD and these syndromes can benefit significantly from the integration of multiple biomarkers. Table 1 displays the markers of APS, their origins, and a comparison with PD.

**Table 1.** Markers of APS, origins, and comparisons with PD.

**Disease**	**Marker**	**Origin cell**	**Comparison in PD and APS**	**Ref**
**MSA**	α-syn	Oligodendrocyte exosomes	Exosomal α-syn: control < PD < MSA	[102],[103]
**PSP**	miRNAs	Serum exosomes	Exosomal miRNAs (miR-21–3p, miR-199a-5p, miR-425–5p, miR-483–5p, miR-22–3p, and miR-29a-3p): PD < PSP	[105]
**DLB**	α-syn	CSF exosomes	Exosomal α-syn: DLB < PD	[46]
**CBD**	Abnormal tau proteins	Neural-Derived exosomes	Exosomal tau proteins: PD < CBD	[112]

## 7. Exosomes in PD: Treatments

Presently, therapeutic interventions for PD do not yield a curative outcome, notwithstanding the existence of several pharmaceutical agents capable of ameliorating motor manifestations. Nevertheless, as the disease progresses, the administration of these pharmaceutical agents may have adverse or deleterious consequences [114]. Hence, there is a pressing demand for the exploration and development of novel pharmaceuticals or methodologies for addressing PD.

Owing to the inability to cross the BBB, the majority of medications used for CNS illnesses have failed in clinical trials [16]. However, exosomes are naturally occurring nanovesicles with the potential to cross the BBB, which makes them appropriate for use as drug delivery vehicles [115]–[117]. Qu et al. separated human blood exosomes and filled them with a saturated dopamine solution. Researchers have shown that these blood-derived exosomes can cross the BBB and transport dopamine to the brain through interactions between transferrin and the transferrin receptor in both in vivo and in vitro trials. Additionally, compared with free dopamine, dopamine-loaded exosomes are less toxic when given systemically via intravenous methods and have improved therapeutic effectiveness in a mouse model of PD [17]. Using monocytes and macrophages, Haney et al. developed a new exosome transport method that includes catalase, a potent antioxidant. In both in vitro and in vivo models of PD, neurons ingest these exosomes and release catalase, which substantially reduces brain inflammation and improves neural survival [18]. Additionally, Kojima et al. reported the creation of a set of modified cells known as exosomal transfer into cells (exotic), which allowed customized exosomes to be produced within altered mammalian cells to transport therapeutic catalase mRNA to the brain [118].

Furthermore, exosomes that carry specific exogenous siRNAs may be used as therapeutic agents for PD. When manufactured exosomes carrying siRNAs targeting α-syn were systemically administered to S129D α-syn transgenic mice, the levels of α-syn mRNA transcription and protein synthesis were reduced [19]. Because siRNAs function only for a short time, Izco and colleagues created shRNA minicircles (shRNA-MCs) [119]. Using RVG-exosomes, shRNA-MCs targeting α-syn were transferred to a mouse model of PD that was produced by premade α-syn fibrils. This intervention improved clinical symptoms, reduced α-syn aggregation, and decreased dopaminergic neuron death.

The utilization of exosomes derived from MSCs has been acknowledged as a potentially beneficial therapeutic resource. Its application has demonstrated benefits in a range of medical disorders, such as PD, MS, and osteoarthritis [120],[121]. Exosomes derived from MSCs have been shown to preserve dopaminergic neurons in 6-hydroxydopamine-induced mouse models of PD, suggesting that they may be therapeutic options for this condition [122]. In animal models of PD, MSC-derived exosomes have been shown to transfer advantageous miRNAs and interact with neuronal cells, resulting in a decrease in neuroinflammation and stimulation of neurogenesis [123]. Notably, miR-21 and miR-143, which are present in MSC-derived exosomes, are essential for immunological control and prevention of neuronal death [124]. Moreover, exosomes produced from MSCs may transmit miR-133b, one of the downregulated miRNAs in PD, to neuronal cells, where it stimulates neurite development [125]. Furthermore, altering MSC-derived exosomes with mimic-miR-7 reduces NLRP3 inflammasome activation and α-syn aggregation in the substantia nigra pars compacta and striatum, which helps to reduce the neuroinflammatory response in PD [124]. Additionally, the addition of antago-miR-155 can decrease neuroinflammation and microglial cell activation, which may have therapeutic effects on PD. The transfer of genetic material, such as miRNAs, via MSC-derived exosomes offers great potential for PD animal models, in light of previously described studies. Understanding how miRNAs from MSC-derived exosomes interact with the molecules and cells implicated in PD is therefore crucial [20].

Exosomes generated from many cell types may be created for specific targeting of neurons and brain regions, as an increasing number of studies have demonstrated. This approach offers therapeutic promise not only for PD but also for many other neurodegenerative illnesses [126]. Although the use of exosomes in therapy has many clear advantages, there are a few drawbacks to consider. First, we were unable to obtain completely pure exosomes using current technologies. As such, designing a simplified exosome delivery system with a minimal amount of superfluous material and a high concentration of therapeutic compounds is critical. Second, a careful examination of the possible negative consequences linked to the use of exosomes from different sources is necessary. Finally, identifying the best biological source for exosomes is important [127].

## 8. Conclusion

In conclusion, this review highlights the significant advancements in exosome research over the past decade, emphasizing their critical role in intercellular communication. These vesicles, which are capable of delivering proteins and miRNAs, influence gene regulation and protein function in recipient cells and facilitate the spread of misfolded proteins such as α-synuclein, contributing to PD pathophysiology. Recent studies underscore the potential of CNS-derived exosomes as diagnostic biomarkers for PD and atypical APS, particularly when they are isolated from specific brain cell types such as astrocytes and oligodendrocytes. Additionally, the unique properties of exosomes, including their ability to cross the blood–brain barrier and their manipulability, make them promising tools for treating neurodegenerative diseases. Further research and development are essential to fully explore and harness the potential of exosomes in the pathogenesis, diagnosis, and treatment of neurodegenerative diseases, especially PD.

## References

[1] (2018). Global, regional, and national burden of Parkinson's disease, 1990–2016: a systematic analysis for the Global Burden of Disease Study 2016. Lancet Neurol.

[2] (2020). Movement Disorders: Volume 35, Number S1, September 2020. Mov Disord.

[3] Ascherio A, Schwarzschild MA (2016). The epidemiology of Parkinson's disease: risk factors and prevention. Lancet Neurol.

[4] Kim CY, Alcalay RN (2017). Genetic Forms of Parkinson's Disease. Semin Neurol.

[5] Dauer W, Przedborski S (2003). Parkinson's disease: mechanisms and models. Neuron.

[6] Braak H, Del Tredici K, Bratzke H (2002). Staging of the intracerebral inclusion body pathology associated with idiopathic Parkinson's disease (preclinical and clinical stages). J Neurol.

[7] Jankovic J (2008). Parkinson's disease: clinical features and diagnosis. J Neurol Neurosurg Psychiatry.

[8] Langston JW (2006). The parkinson's complex: Parkinsonism is just the tip of the iceberg. Ann Neurol.

[9] Postuma RB, Berg D, Stern M (2015). MDS clinical diagnostic criteria for Parkinson's disease. Mov Disord.

[10] Langston JW (1990). Predicting Parkinson's disease. Neurology.

[11] Braak H, Del Tredici K, Rüb U (2003). Staging of brain pathology related to sporadic Parkinson's disease. Neurobiol Aging.

[12] Bellingham S, Guo B, Coleman B (2012). Exosomes: Vehicles for the Transfer of Toxic Proteins Associated with Neurodegenerative Diseases?. Front Physiol.

[13] Guo M, Wang J, Zhao Y (2020). Microglial exosomes facilitate α-synuclein transmission in Parkinson's disease. Brain.

[14] Yang T, Martin P, Fogarty B (2015). Exosome delivered anticancer drugs across the blood-brain barrier for brain cancer therapy in Danio rerio. Pharm Res.

[15] Goetzl EJ, Boxer A, Schwartz JB (2015). Altered lysosomal proteins in neural-derived plasma exosomes in preclinical Alzheimer disease. Neurology.

[16] Pardridge WM (2012). Drug transport across the blood–brain barrier. J Cerebr Blood F Met.

[17] Qu M, Lin Q, Huang L (2018). Dopamine-loaded blood exosomes targeted to brain for better treatment of Parkinson's disease. J Control Release.

[18] Haney MJ, Klyachko NL, Zhao Y (2015). Exosomes as drug delivery vehicles for Parkinson's disease therapy. J Control Release.

[19] Cooper JM, Wiklander PO, Nordin JZ (2014). Systemic exosomal siRNA delivery reduced alpha-synuclein aggregates in brains of transgenic mice. Mov Disord.

[20] Guy R, Offen D (2020). Promising opportunities for treating neurodegenerative diseases with mesenchymal stem cell-derived exosomes. Biomolecules.

[21] Théry C, Zitvogel L, Amigorena S (2002). Exosomes: composition, biogenesis and function. Nat Rev Immunol.

[22] Akbari-Gharalari N, Khodakarimi S, Nezhadshahmohammad F (2024). Exosomes in neuron-glia communication: A review on neurodegeneration. Bioimpacts.

[23] Kalluri R, LeBleu VS (2020). The biology, function, and biomedical applications of exosomes. Science.

[24] Omrani M, Beyrampour-Basmenj H, Jahanban-Esfahlan R (2023). Global trend in exosome isolation and application: an update concept in management of diseases. Mol Cell Biochem.

[25] Lachenal G, Pernet-Gallay K, Chivet M (2011). Release of exosomes from differentiated neurons and its regulation by synaptic glutamatergic activity. Mol Cell Neurosci.

[26] Wubbolts R, Leckie RS, Veenhuizen PT (2003). Proteomic and biochemical analyses of human B cell-derived exosomes. Potential implications for their function and multivesicular body formation. J Biol Chem.

[27] van Meer G, Voelker DR, Feigenson GW (2008). Membrane lipids: where they are and how they behave. Nat Rev Mol Cell Biol.

[28] Zhang Y, Bi J, Huang J (2020). Exosome: A Review of Its Classification, Isolation Techniques, Storage, Diagnostic and Targeted Therapy Applications. Int J Nanomedicine.

[29] Skokos D, Le Panse S, Villa I (2001). Mast cell-dependent B and T lymphocyte activation is mediated by the secretion of immunologically active exosomes. J Immunol.

[30] Chen G, Huang AC, Zhang W (2018). Exosomal PD-L1 contributes to immunosuppression and is associated with anti-PD-1 response. Nature.

[31] Valadi H, Ekström K, Bossios A (2007). Exosome-mediated transfer of mRNAs and microRNAs is a novel mechanism of genetic exchange between cells. Nat Cell Biol.

[32] Sharma A, Johnson A (2020). Exosome DNA: Critical regulator of tumor immunity and a diagnostic biomarker. J Cell Physiol.

[33] Harding CV, Heuser JE, Stahl PD (2013). Exosomes: looking back three decades and into the future. Cell Biol.

[34] Pravin DP, Aashutosh US (2016). Molecular Biomarkers for Diagnosis & Therapies of Alzheimer's Disease. AIMS Neurosci.

[35] Yakubovich E, Polischouk A, Evtushenko V (2022). Principles and problems of exosome isolation from biological fluids. Biochem Mosc Suppl S.

[36] Kučuk N, Primožič M, Knez Ž (2021). Exosomes engineering and their roles as therapy delivery tools, therapeutic targets, and biomarkers. Int J Mol Sci.

[37] Budnik V, Ruiz-Cañada C, Wendler F (2016). Extracellular vesicles round off communication in the nervous system. Nat Rev Neurosci.

[38] Desplats P, Lee HJ, Bae EJ (2009). Inclusion formation and neuronal cell death through neuron-to-neuron transmission of alpha-synuclein. Proc Natl Acad Sci U S A.

[39] Crews L, Spencer B, Desplats P (2010). Selective Molecular Alterations in the Autophagy Pathway in Patients with Lewy Body Disease and in Models of α-Synucleinopathy. PLOS ONE.

[40] Minakaki G, Menges S, Kittel A (2018). Autophagy inhibition promotes SNCA/alpha-synuclein release and transfer via extracellular vesicles with a hybrid autophagosome-exosome-like phenotype. Autophagy.

[41] Delenclos M, Trendafilova T, Mahesh D (2017). Investigation of Endocytic Pathways for the Internalization of Exosome-Associated Oligomeric Alpha-Synuclein. Front Neurosci.

[42] Huang Y, Liu Z, Li N (2022). Parkinson's Disease Derived Exosomes Aggravate Neuropathology in SNCA*A53T Mice. Ann Neurol.

[43] Han C, Xiong N, Guo X (2019). Exosomes from patients with Parkinson's disease are pathological in mice. J Mol Med (Berl).

[44] Sano K, Atarashi R, Satoh K (2018). Prion-Like Seeding of Misfolded α-Synuclein in the Brains of Dementia with Lewy Body Patients in RT-QUIC. Mol Neurobiol.

[45] Danzer KM, Kranich LR, Ruf WP (2012). Exosomal cell-to-cell transmission of alpha synuclein oligomers. Mol Neurodegener.

[46] Stuendl A, Kunadt M, Kruse N (2016). Induction of α-synuclein aggregate formation by CSF exosomes from patients with Parkinson's disease and dementia with Lewy bodies. Brain.

[47] Herman S, Djaldetti R, Mollenhauer B (2022). CSF-derived extracellular vesicles from patients with Parkinson's disease induce symptoms and pathology. Brain.

[48] Yakabi K, Berson E, Montine KS (2023). Human cerebrospinal fluid single exosomes in Parkinson's and Alzheimer's diseases. bioRxiv.

[49] Shi M, Liu C, Cook TJ (2014). Plasma exosomal α-synuclein is likely CNS-derived and increased in Parkinson's disease. Acta Neuropathol.

[50] Kenwrick S, Watkins A, Angelis ED (2000). Neural cell recognition molecule L1: relating biological complexity to human disease mutations. Hum Mol Genet.

[51] Niu M, Li Y, Li G (2020). A longitudinal study on α-synuclein in plasma neuronal exosomes as a biomarker for Parkinson's disease development and progression. Eur J Neurol.

[52] Yan YQ, Pu JL, Zheng R (2022). Different patterns of exosomal α-synuclein between Parkinson's disease and probable rapid eye movement sleep behavior disorder. Eur J Neurol.

[53] Postuma RB, Gagnon JF, Bertrand JA (2015). Parkinson risk in idiopathic REM sleep behavior disorder: preparing for neuroprotective trials. Neurology.

[54] Shim KH, Go HG, Bae H (2021). Decreased Exosomal Acetylcholinesterase Activity in the Plasma of Patients With Parkinson's Disease. Front Aging Neurosci.

[55] Si X, Tian J, Chen Y (2019). Central Nervous System-Derived Exosomal Alpha-Synuclein in Serum May Be a Biomarker in Parkinson's Disease. Neuroscience.

[56] Kluge A, Bunk J, Schaeffer E (2022). Correction to: Detection of neuron-derived pathological α-synuclein in blood. Brain.

[57] Zheng H, Xie Z, Zhang X (2021). Investigation of α-Synuclein Species in Plasma Exosomes and the Oligomeric and Phosphorylated α-Synuclein as Potential Peripheral Biomarker of Parkinson's Disease. Neuroscience.

[58] Zhentang C, Yufeng W, Genliang L (2019). α-Synuclein in salivary extracellular vesicles as a potential biomarker of Parkinson's disease. Neurosci Lett.

[59] Rani K, Mukherjee R, Singh E (2019). Neuronal exosomes in saliva of Parkinson's disease patients: A pilot study. Parkinsonism Relat D.

[60] Kang W, Chen W, Yang Q (2016). Salivary total α-synuclein, oligomeric α-synuclein and SNCA variants in Parkinson's disease patients. Sci Rep.

[61] Shu H, Zhang P, Gu L (2024). Alpha-synuclein in peripheral body fluid as a biomarker for Parkinson's disease. Acta Neurol Belg.

[62] Clements CM, McNally RS, Conti BJ (2006). DJ-1, a cancer- and Parkinson's disease-associated protein, stabilizes the antioxidant transcriptional master regulator Nrf2. Proc Natl Acad Sci U S A.

[63] Jang J, Jeong S, Lee SI (2018). Oxidized DJ-1 levels in urine samples as a putative biomarker for Parkinson's disease. Parkinsons Dis.

[64] An C, Pu X, Xiao W (2018). Expression of the DJ-1 protein in the serum of Chinese patients with Parkinson's disease. Neurosci Lett.

[65] Waragai M, Nakai M, Wei J (2007). Plasma levels of DJ-1 as a possible marker for progression of sporadic Parkinson's disease. Neurosci Lett.

[66] T. dos Santos MC, Scheller D, Schulte C (2018). Evaluation of cerebrospinal fluid proteins as potential biomarkers for early stage Parkinson's disease diagnosis. PLOS ONE.

[67] Zhao Z-H, Chen Z-T, Zhou R-L (2019). Increased DJ-1 and α-synuclein in plasma neural-derived exosomes as potential markers for Parkinson's disease. Front Aging Neurosci.

[68] Gonzales PA, Pisitkun T, Hoffert JD (2009). Large-Scale Proteomics and Phosphoproteomics of Urinary Exosomes. J Am Soc Nephrol.

[69] Pisitkun T, Shen R-F, Knepper MA (2004). Identification and proteomic profiling of exosomes in human urine. P Natl Acad Sci.

[70] Ho DH, Yi S, Seo H (2014). Increased DJ-1 in urine exosome of Korean males with Parkinson's disease. Biomed Res Int.

[71] Goedert M, Spillantini MG (2006). A century of Alzheimer's disease. Science.

[72] Spillantini MG, Goedert M (2013). Tau pathology and neurodegeneration. Lancet Neurol.

[73] Wills J, Jones J, Haggerty T (2010). Elevated tauopathy and alpha-synuclein pathology in postmortem Parkinson's disease brains with and without dementia. Exp Neurol.

[74] Edwards TL, Scott WK, Almonte C (2010). Genome-wide association study confirms SNPs in SNCA and the MAPT region as common risk factors for Parkinson disease. Ann Hum Genet.

[75] Simón-Sánchez J, Schulte C, Bras JM (2009). Genome-wide association study reveals genetic risk underlying Parkinson's disease. Nat Genet.

[76] Vermilyea SC, Christensen A, Meints J (2022). Loss of tau expression attenuates neurodegeneration associated with α-synucleinopathy. Transl Neurodegener.

[77] Shi M, Kovac A, Korff A (2016). CNS tau efflux via exosomes is likely increased in Parkinson's disease but not in Alzheimer's disease. Alzheimers Dement.

[78] Berg D, Schweitzer KJ, Leitner P (2005). Type and frequency of mutations in the LRRK2 gene in familial and sporadic Parkinson's disease*. Brain.

[79] Paisán-Ruíz C, Jain S, Evans EW (2004). Cloning of the gene containing mutations that cause PARK8-linked Parkinson's disease. Neuron.

[80] Rosenbusch KE, Kortholt A (2016). Activation Mechanism of LRRK2 and Its Cellular Functions in Parkinson's Disease. Parkinsons Dis.

[81] Fraser KB, Moehle MS, Daher JP (2013). LRRK2 secretion in exosomes is regulated by 14-3-3. Hum Mol Genet.

[82] Fraser KB, Moehle MS, Alcalay RN (2016). Urinary LRRK2 phosphorylation predicts parkinsonian phenotypes in G2019S LRRK2 carriers. Neurology.

[83] Di Maio R, Hoffman EK, Rocha EM (2018). LRRK2 activation in idiopathic Parkinson's disease. Sci Transl Med.

[84] Fraser KB, Rawlins AB, Clark RG (2016). Ser(P)-1292 LRRK2 in urinary exosomes is elevated in idiopathic Parkinson's disease. Mov Disord.

[85] Wang H (2021). MicroRNAs, Parkinson's Disease, and Diabetes Mellitus. Int J Mol Sci.

[86] Gui Y, Liu H, Zhang L (2015). Altered microRNA profiles in cerebrospinal fluid exosome in Parkinson disease and Alzheimer disease. Oncotarget.

[87] Nie C, Sun Y, Zhen H (2020). Differential Expression of Plasma Exo-miRNA in Neurodegenerative Diseases by Next-Generation Sequencing. Front Neurosci.

[88] Yao YF, Qu MW, Li GC (2018). Circulating exosomal miRNAs as diagnostic biomarkers in Parkinson's disease. Eur Rev Med Pharmacol Sci.

[89] Margis R, Margis R, Rieder CR (2011). Identification of blood microRNAs associated to Parkinsonĭs disease. J Biotechnol.

[90] Botta-Orfila T, Morató X, Compta Y (2014). Identification of blood serum micro-RNAs associated with idiopathic and LRRK2 Parkinson's disease. J Neurosci Res.

[91] Vallelunga A, Ragusa M, Di Mauro S (2014). Identification of circulating microRNAs for the differential diagnosis of Parkinson's disease and Multiple System Atrophy. Front Cell Neurosci.

[92] Cao XY, Lu JM, Zhao ZQ (2017). MicroRNA biomarkers of Parkinson's disease in serum exosome-like microvesicles. Neurosci Lett.

[93] Qian X, Zhao J, Yeung PY (2019). Revealing lncRNA Structures and Interactions by Sequencing-Based Approaches. Trends Biochem Sci.

[94] Wu P, Zuo X, Deng H (2013). Roles of long noncoding RNAs in brain development, functional diversification and neurodegenerative diseases. Brain Res Bull.

[95] Zou J, Guo Y, Wei L (2020). Long Noncoding RNA POU3F3 and α-Synuclein in Plasma L1CAM Exosomes Combined with β-Glucocerebrosidase Activity: Potential Predictors of Parkinson's Disease. Neurotherapeutics.

[96] Boer DEC, van Smeden J, Bouwstra JA (2020). Glucocerebrosidase: Functions in and Beyond the Lysosome. J Clin Med.

[97] Fanciulli A, Wenning GK (2015). Multiple-system atrophy. N Engl J Med.

[98] Gilman S, Wenning GK, Low PA (2008). Second consensus statement on the diagnosis of multiple system atrophy. Neurology.

[99] Fanciulli A, Stankovic I, Krismer F (2019). Multiple system atrophy. Int Rev Neurobiol.

[100] Galvin JE, Lee VM, Trojanowski JQ (2001). Synucleinopathies: clinical and pathological implications. Arch Neurol.

[101] Wenning GK, Ben-Shlomo Y, Hughes A (2000). What clinical features are most useful to distinguish definite multiple system atrophy from Parkinson's disease?. J Neurol Neurosurg Psychiatry.

[102] Jiang C, Hopfner F, Katsikoudi A (2020). Serum neuronal exosomes predict and differentiate Parkinson's disease from atypical parkinsonism. J Neurol Neurosurg Psychiatry.

[103] Dutta S, Hornung S, Kruayatidee A (2021). α-Synuclein in blood exosomes immunoprecipitated using neuronal and oligodendroglial markers distinguishes Parkinson's disease from multiple system atrophy. Acta Neuropathol.

[104] Höglinger GU, Respondek G, Stamelou M (2017). Clinical diagnosis of progressive supranuclear palsy: The movement disorder society criteria. Mov Disord.

[105] Manna I, Quattrone A, De Benedittis S (2021). Exosomal miRNA as peripheral biomarkers in Parkinson's disease and progressive supranuclear palsy: A pilot study. Parkinsonism Relat Disord.

[106] McKeith IG, Dickson DW, Lowe J (2005). Diagnosis and management of dementia with Lewy bodies: third report of the DLB Consortium. Neurology.

[107] Lippa CF, Duda JE, Grossman M (2007). DLB and PDD boundary issues. Diagnosis Treatment Molecular Pathology Biomarkers.

[108] Bonanni L, Thomas A, Onofrj M (2006). Diagnosis and management of dementia with Lewy bodies: third report of the DLB Consortium. Neurology.

[109] Emre M, Aarsland D, Brown R (2007). Clinical diagnostic criteria for dementia associated with Parkinson's disease. Mov Disord.

[110] Walker Z, Possin KL, Boeve BF (2015). Lewy body dementias. Lancet.

[111] Swallow DMA, Counsell CE (2023). The evolution of diagnosis from symptom onset to death in progressive supranuclear palsy (PSP) and corticobasal degeneration (CBD) compared to Parkinson's disease (PD). J Neurol.

[112] Meloni M, Agliardi C, Guerini FR (2023). Oligomeric α-synuclein and tau aggregates in NDEVs differentiate Parkinson's disease from atypical parkinsonisms. Neurobiol Dis.

[113] Jiang C, Hopfner F, Berg D (2021). Validation of α-Synuclein in L1CAM-Immunocaptured Exosomes as a Biomarker for the Stratification of Parkinsonian Syndromes. Mov Disord.

[114] Müller T (2012). Drug therapy in patients with Parkinson's disease. Translational Neurodegener.

[115] Zhuang X, Xiang X, Grizzle W (2011). Treatment of brain inflammatory diseases by delivering exosome encapsulated anti-inflammatory drugs from the nasal region to the brain. Mol Ther.

[116] Lai CP-K, Breakefield XO (2012). Role of exosomes/microvesicles in the nervous system and use in emerging therapies. Front Physiol.

[117] Akbari-Gharalari N, Ghahremani-Nasab M, Naderi R (2023). Improvement of spinal cord injury symptoms by targeting the Bax/Bcl2 pathway and modulating TNF-α/IL-10 using Platelet-Rich Plasma exosomes loaded with dexamethasone. AIMS Neurosci.

[118] Kojima R, Bojar D, Rizzi G (2018). Designer exosomes produced by implanted cells intracerebrally deliver therapeutic cargo for Parkinson's disease treatment. Nat Commun.

[119] Izco M, Blesa J, Schleef M (2019). Systemic exosomal delivery of shRNA minicircles prevents parkinsonian pathology. Mol Ther.

[120] Mianehsaz E, Mirzaei HR, Mahjoubin-Tehran M (2019). Mesenchymal stem cell-derived exosomes: a new therapeutic approach to osteoarthritis?. Stem Cell Res Ther.

[121] Li Z, Liu F, He X (2019). Exosomes derived from mesenchymal stem cells attenuate inflammation and demyelination of the central nervous system in EAE rats by regulating the polarization of microglia. Int Immunopharmacol.

[122] Vilaça-Faria H, Salgado AJ, Teixeira FG (2019). Mesenchymal stem cells-derived exosomes: a new possible therapeutic strategy for Parkinson's disease?. Cells.

[123] Heris RM, Shirvaliloo M, Abbaspour-Aghdam S (2022). The potential use of mesenchymal stem cells and their exosomes in Parkinson's disease treatment. Stem Cell Res Ther.

[124] Abrishamdar M, Jalali MS, Yazdanfar N (2023). The role of exosomes in pathogenesis and the therapeutic efficacy of mesenchymal stem cell-derived exosomes against Parkinson's disease. Neurol Sci.

[125] Kattaia A, Abd EL-Baset S, Abdul-Maksoud R (2022). The therapeutic potential of exosomes derived from mesenchymal stem cells in experimentally induced hypertensive encephalopathy. J Med Histol.

[126] Tomlinson PR, Zheng Y, Fischer R (2015). Identification of distinct circulating exosomes in Parkinson's disease. Ann Clin Transl Neur.

[127] Sarko DK, McKinney CE (2017). Exosomes: origins and therapeutic potential for neurodegenerative disease. Front Neurosci.

